# Targeting a splicing-mediated drug resistance mechanism in prostate cancer by inhibiting transcriptional regulation by PKCβ1

**DOI:** 10.1038/s41388-022-02179-z

**Published:** 2022-01-27

**Authors:** James E. Melnyk, Veronica Steri, Hao G. Nguyen, Y. Christina Hwang, John D. Gordan, Byron Hann, Felix Y. Feng, Kevan M. Shokat

**Affiliations:** 1 Department of Cellular and Molecular Pharmacology, University of California, San Francisco, San Francisco, CA 94158 USA; 2 Helen Diller Family Comprehensive Cancer Center, University of California, San Francisco, San Francisco, CA 94158 USA; 3 Preclinical Therapeutics Core, University of California San Francisco, San Francisco, CA 94158 USA; 4 Department of Urology, University of California, San Francisco, San Francisco, CA 94143 USA; 5 Department of Medicine and Division of Hematology/Oncology, University of California, San Francisco, San Francisco, CA 94158 USA; 6 Department of Radiation Oncology, University of California, San Francisco, San Francisco, CA 94143 USA; 7 Department of Medicine, University of California, San Francisco, San Francisco, CA 94143 USA; 8 Howard Hughes Medical Institute, University of California, San Francisco, San Francisco, CA 94143 USA

**Keywords:** Prostate cancer, Mechanisms of disease, Transcription

## Abstract

The androgen receptor (AR) is a central driver of aggressive prostate cancer. After initial treatment with androgen receptor signaling inhibitors (ARSi), reactivation of AR signaling leads to resistance. Alternative splicing of AR mRNA yields the AR-V7 splice variant, which is currently an undruggable mechanism of ARSi resistance: AR-V7 lacks a ligand binding domain, where hormones and anti-androgen antagonists act, but still activates AR signaling. We reveal PKCβ as a druggable regulator of transcription and splicing at the AR genomic locus. We identify a clinical PKCβ inhibitor in combination with an FDA-approved anti-androgen as an approach for repressing AR genomic locus expression, including expression of AR-V7, while antagonizing full-length AR. PKCβ inhibition reduces total AR gene expression, thus reducing AR-V7 protein levels and sensitizing prostate cancer cells to current anti-androgen therapies. We demonstrate that this combination may be a viable therapeutic strategy for AR-V7-positive prostate cancer.

## Introduction

Androgen receptor signaling inhibitors (ARSi) are currently the primary treatment regimen for advanced prostate cancer. These therapies work either by directly antagonizing the AR at its ligand-binding domain (LBD) or by inhibiting androgen synthesis. Such treatments are generally initially successful, but many patients eventually relapse and develop lethal, metastatic castration-resistant prostate cancer (CRPC), which thrives even in a reduced-hormone environment [1]. CRPC progresses through several possible mechanisms, including complete AR independence, LBD mutations that relax steroid-binding specificity, adrenal or intra-tumoral androgen synthesis, amplification of the AR gene body and its enhancers, and the AR-V7 alternative splice variant [1–4].

The presence of the AR-V7 splice variant is associated with resistance to ARSi therapies and clinically poor outcomes [3]. AR-V7 is a constitutively active, androgen-independent transcription factor that lacks its LBD but retains its DNA-binding domain and is thus able to circumvent the actions of current anti-androgen therapies that target the LBD [5–7]. Elegant prior work reveals that AR expression increases during androgen blockade and concomitantly leads to AR alternative splicing and production of AR-V7 [5–9]. While anti-androgen therapies block activation of full-length AR, AR-V7 which is also produced lacks the LBD resulting in an undruggable isoform of the druggable AR oncogene [5, 7], and has established an unmet need for novel therapeutic approaches to target AR-V7.

Targeting AR-V7 is currently an active and dynamic area of drug discovery. Drugs with several conceptually distinct approaches have been identified and pursued: (1) small molecules that bind to the AR-V7 protein despite its lack of known small-molecule-targetable features [10–13], (2) small molecules which lead to degradation of AR-V7 [14–16], (3) agents that act indirectly to target AR-V7 [17, 18], and (4) antisense oligonucleotides and small molecules that modulate AR pre-mRNA alternative splicing [19–22]. To date, efforts to target AR pre-mRNA in prostate cancer have been dominated by antisense oligonucleotide strategies that appear promising but have yielded very limited clinical success [19–21, 23, 24]. We were therefore encouraged to evaluate a small molecule approach to target transcription and splicing of AR pre-mRNA in AR-V7-positive prostate cancer.

The AR has a well-established role as an androgen-dependent transcriptional activator, and the mechanistic details for the activation of its targets are well studied [25–29]. But importantly, the AR is also responsible for androgen-dependent transcriptional repression. In its transcriptional suppressor role, the androgen-bound full-length AR recruits chromatin-modifying complexes to genomic targets to remove activating histone marks and recruits transcription suppressors [29, 30]. Genes repressed by the AR notably include the AR itself; in a high-androgen environment, the androgen-bound full-length AR inhibits transcription at the AR genomic locus by binding repressive sites along intron 2, causing a reduction in total AR mRNA transcripts in a negative feedback loop [6, 31]. Conversely, anti-androgen therapies such as enzalutamide (MDV) block androgen-binding and localization to the AR genomic locus, resulting in increased transcriptional activity at the AR locus and an increase in total AR mRNA transcripts, including those of the AR-V7 splice variant that drives resistance to the same therapies. We hypothesized that druggable kinases may regulate transcription and splicing at the AR genomic locus to mediate the changes in AR gene expression observed in CRPC.

On the basis of this hypothesis, we evaluated potential kinase targets and selected the protein kinase PKCβ1 for investigation. PKCβ1 is reported to be associated with active transcription in prostate cancer and phosphorylates histone H3T6 [27]. H3T6 phosphorylation blocks lysine demethylases from removing mono- and dimethyl marks at H3K4, preventing transcriptional repression [27]. We, therefore, hypothesized that PKCβ1 could be present at the AR genomic locus during androgen blockade and promoting transcription by phosphorylating H3T6. In this study, we investigate the use of PKCβ inhibition to reduce anti-androgen-driven transcriptional activation at the AR genomic locus. We find that inhibiting PKCβ reduces total AR transcript levels, including AR-V7 splice variant levels, and sensitizes AR-V7-positive prostate cancer cells to existing anti-androgen therapies.

## Results

### Androgen negatively regulates AR gene expression in VCaP cells

The VCaP prostate cancer cell line is a metastatic CRPC model that expresses full-length wild-type (WT) AR and the alternatively spliced AR-V7 isoform [5, 7–9]. This cell line has an amplification of the AR locus and is androgen-responsive [32]. When VCaP cells are cultured in growth medium supplemented with charcoal-stripped serum (CSS), a low-androgen environment, full-length AR and AR-V7 mRNA and protein levels are elevated [5, 7–9]. Conversely, after treatment for 24 h with the AR agonist dihydrotestosterone (DHT), full-length AR and AR-V7 mRNA and protein levels are markedly reduced in a dose-dependent manner (Supplementary Fig. S1A and S1B; Supplementary Table S1).

### PKCβ1 expression increases during androgen blockade

**Fig. 1:**
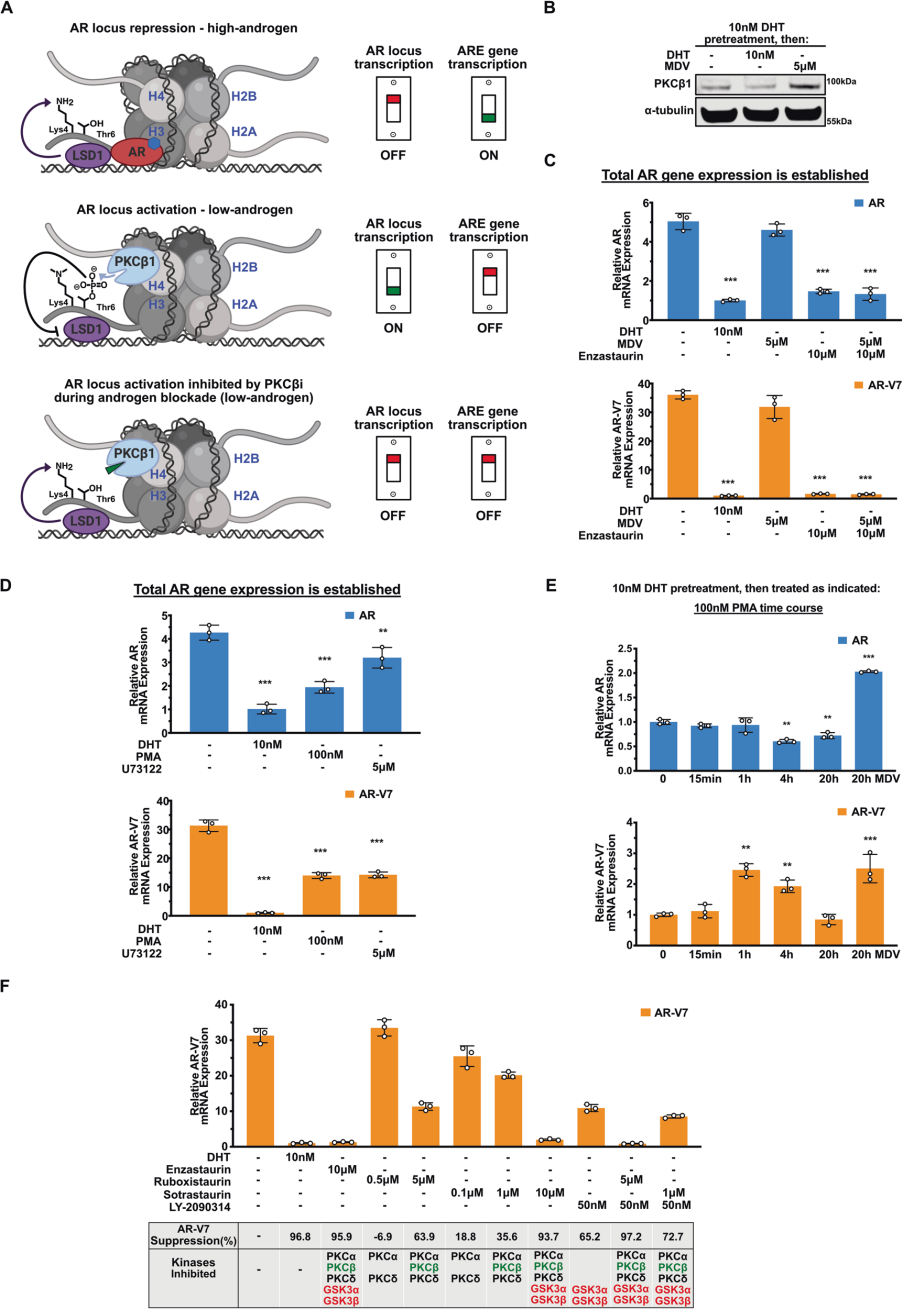
PKCβ1 inhibition reduces expression of the AR genomic locus and decreases AR and AR-V7 mRNA transcripts. **A** Images of a nucleosome with each of the four core histone proteins indicated. Established mechanism of AR locus repression in high-androgen conditions (top). Proposed mechanism of AR locus activation by PKCβ1 in low-androgen conditions (middle). Proposed inhibition of AR locus activation during androgen blockade by a PKCβ1 inhibitor (bottom). The blue hexagon represents DHT and the green triangle represents a PKCβ1 inhibitor. **B** VCaP cells cultured in RPMI1640 supplemented with 5% CSS for 48 h were treated for 24 h with DHT, washed out, then treated as indicated for an additional 24 h and immunoblotted for PKCβ1. **C, D** VCaP cells cultured in RPMI1640 supplemented with 5% CSS for 48 h were treated in three biological replicates for 24 h as indicated and analyzed by RT-qPCR for AR and AR-V7 mRNA transcript levels. Data are mean ± SD. P-values are relative to vehicle (***p-value < 0.0001). All mRNA expression levels are relative to GAPDH and normalized to the response for DHT. **E** VCaP cells cultured in RPMI1640 supplemented with 5% CSS for 48 h, pre-treated with 10 nM DHT for 24 h, and then washed out and treated with 100 nM PMA for the indicated timepoints, or with 5 μM MDV for 20 h and analyzed by RT-qPCR for AR and AR-V7 mRNA transcript levels. Data are mean ± SD. P-values are relative to time 0 (**p-value < 0.005; ***p-value < 0.0001). All mRNA expression levels are relative to GAPDH and normalized to the response for time 0. **F** VCaP cells cultured in RPMI1640 supplemented with 5% CSS for 48 h were treated in three biological replicates for 24 h with PKC and GSK3 inhibitors and analyzed by RT-qPCR for AR-V7 mRNA transcript levels. Table shows % suppression of AR-V7 mRNA transcripts relative to vehicle. Data are mean ± SD. All mRNA expression levels are relative to GAPDH and normalized to the response for DHT.

Prior work revealed that prolonged exposure to high-androgen environments leads to binding of full-length AR at repressive sites along intron 2 of the AR genomic locus, which recruits the lysine demethylase LSD1 (KDM1A) to remove methyl marks from histone H3K4 as represented in Fig. 1A**(top)**, suppressing AR gene expression [6]. The protein kinase PKCβ1 is reported to promote active transcription by phosphorylating histone H3T6, which blocks LSD1 demethylase activity at H3K4 [27]. We, therefore, hypothesized that PKCβ1 is present at the AR genomic locus during low-androgen conditions, promoting transcription and increasing total AR transcript levels by phosphorylating histone H3T6 **(**Fig. 1A**(middle))**. We assessed PKCβ1 protein expression in the VCaP cell line and found that it is downregulated by DHT and upregulated by MDV (Fig. 1B). This observation aligns with a prior report that MDV induces PKC-family members in prostate cancer cells [33]. We propose PKCβ1 as an important component of the low-androgen stress response that upregulates AR gene expression and increases full-length AR and AR-V7 protein levels during AR antagonism.

### Enzastaurin reduces AR and AR-V7 mRNA transcript levels

We hypothesized that inhibition of PKCβ1-mediated H3T6 phosphorylation would allow demethylation of H3K4, thus decreasing transcriptional activity at the AR genomic locus **(**Fig. 1A**(bottom))**, and consequently decreasing both AR and AR-V7 mRNA transcripts. We assessed the ability of the PKCβ inhibitor enzastaurin to suppress both full-length AR and AR-V7 mRNA transcripts. VCaP cells were cultured for 48 h in a low-androgen environment to maximize the low-androgen stress response that increases transcription at the AR genomic locus [5, 7–9]. Cells were subsequently treated with either MDV, enzastaurin, or both **(**Fig. 1C**)**. This assay is designed to mimic a clinical situation in which prostate cancer is highly expressing full-length AR and AR-V7. As hypothesized, enzastaurin alone or in combination with MDV reduced the level of both full-length AR and AR-V7 mRNA transcripts. Full-length AR transcripts decreased by 3.4-fold and 3.9-fold respectively relative to the vehicle, while AR-V7 mRNA transcripts decreased more starkly by 22.2-fold and 24.5-fold respectively.

We subsequently assessed the ability of enzastaurin to suppress full-length AR and AR-V7 mRNA transcripts while the low-androgen stress response is building, and therefore transcription at the AR genomic locus is increasing. In this assay, VCaP cells were pre-treated with DHT to suppress total AR mRNA transcripts. DHT was subsequently washed out, and the cells were treated with either additional DHT, MDV, enzastaurin, or enzastaurin in combination with MDV. In these conditions, androgen blockade by MDV alleviates androgen-dependent AR genomic locus repression causing an increase in transcriptional activity at the AR genomic locus [5, 6]. This assay is designed to resemble a clinical situation in which the AR-V7 splice variant is emerging during anti-androgen therapy. Enzastaurin was able to reduce MDV-induced expression of full-length AR mRNA transcripts by 1.9-fold and AR-V7 mRNA transcripts by 4.9-fold, relative to MDV **(**Supplementary Fig. S2A). Collectively, the data demonstrate that enzastaurin reduces total AR mRNA transcripts, but with a greater magnitude effect for AR-V7.

### Enzastaurin activity is mediated through PKCβ inhibition

Enzastaurin was developed as a clinical PKCβ inhibitor, however, it also exhibits potent inhibition of PKCα, PKCδ, GSK3α, and GSK3β [34]. First, to test whether the effects of enzastaurin on AR transcription are driven by PKC-family kinases, we assessed AR gene expression after chemical knockdown of PKC-family kinases with phorbol 12-myristate 13-acetate (PMA), or after treatment with the phospholipase C inhibitor, U73122, to respectively degrade PKC-family kinases or to suppress second messenger synthesis that activates PKC-family kinases. PMA activates both conventional and novel PKC-family kinases, which leads to their rapid degradation **(**Supplementary Fig. S2B) [35–38]. These kinases have cellular half-lives on the order of days in tissue culture conditions, making genetic knockdown difficult since PKC-family protein levels persist even with successful gene silencing. The use of PMA, therefore, circumvents difficulties with genetic knockdown [37]. U73122 is an inhibitor of Phospholipase C, which hydrolyzes phosphatidylinositol 4,5-bisphosphate (PIP2) to produce diacylglycerol (DAG) and inositol 1,4,5-trisphosphate (IP3), which stimulates the release of Ca^2+^. Both DAG and Ca^2+^ are second messengers for conventional and novel PKC family member activation [39]. Treatment with either PMA or U73122 decreased AR-V7 mRNA transcript levels **(**Fig. 1D**)**. Due to the initial, robust activation of PKC family members induced by PMA prior to degradation, we also performed a time-course experiment to determine if PMA treatment initially increases AR-V7 mRNA transcripts at shorter time points. Our results reveal an increase in AR-V7 mRNA transcript levels after one to four hours. Importantly, in this assay the PMA-induced increase is similar to that observed for MDV. However, an increase in full-length AR mRNA levels is not observed at shorter PMA treatment time points, and a reduction in full-length AR mRNA levels is observed after four hours (Fig. 1E).

We then evaluated the dose-dependent effects of ruboxistaurin and sotrastaurin, PKC-family inhibitors with differing affinities for PKCα, PKCβ, and PKCδ [34], on AR-V7 mRNA transcript levels. We found that inhibitor doses at which PKCβ should be inhibited reduced AR-V7 mRNA levels, while PKCα and PKCδ inhibition alone had little effect **(**Fig. 1F**)**. Additionally, we analyzed LY-2090314 a potent GSK3α and GSK3β inhibitor, and found GSK3α and GSK3β inhibition also yields a partial reduction in AR-V7 mRNA transcript levels. However, the most potent reductions of AR-V7 mRNA levels are observed in conditions when PKCβ, GSK3α, and GSK3β are all simultaneously inhibited (10 μM enzastaurin, 10 μM sotrastaurin, 5 μM ruboxistaurin + 50 nM LY-2090314, and 1 μM sotrastaurin + 50 nM LY-2090314) (Fig. 1F). We then used a multiplexed inhibitor bead (MIB) column strategy [40] to compare kinase activity profiles during MDV or DHT treatment. We found that GSK3α, GSK3β, and several Ca^2+^-stimulated kinases are activated during androgen blockade. We were not able to detect PKCβ1 in this assay (Supplementary Fig. S2C), although this may be due to a low expression level and the presence of other PKC-family members that make detection difficult. In summary, we conclude that the effect of enzastaurin on the AR is primarily mediated by PKCβ inhibition, but is enhanced by its poly-pharmacology against GSK3α and GSK3β.

### PKCβ inhibition reduces histone H3T6 phosphorylation and decreases histone H3K4 methylation at the AR genomic locus

**Fig. 2:**
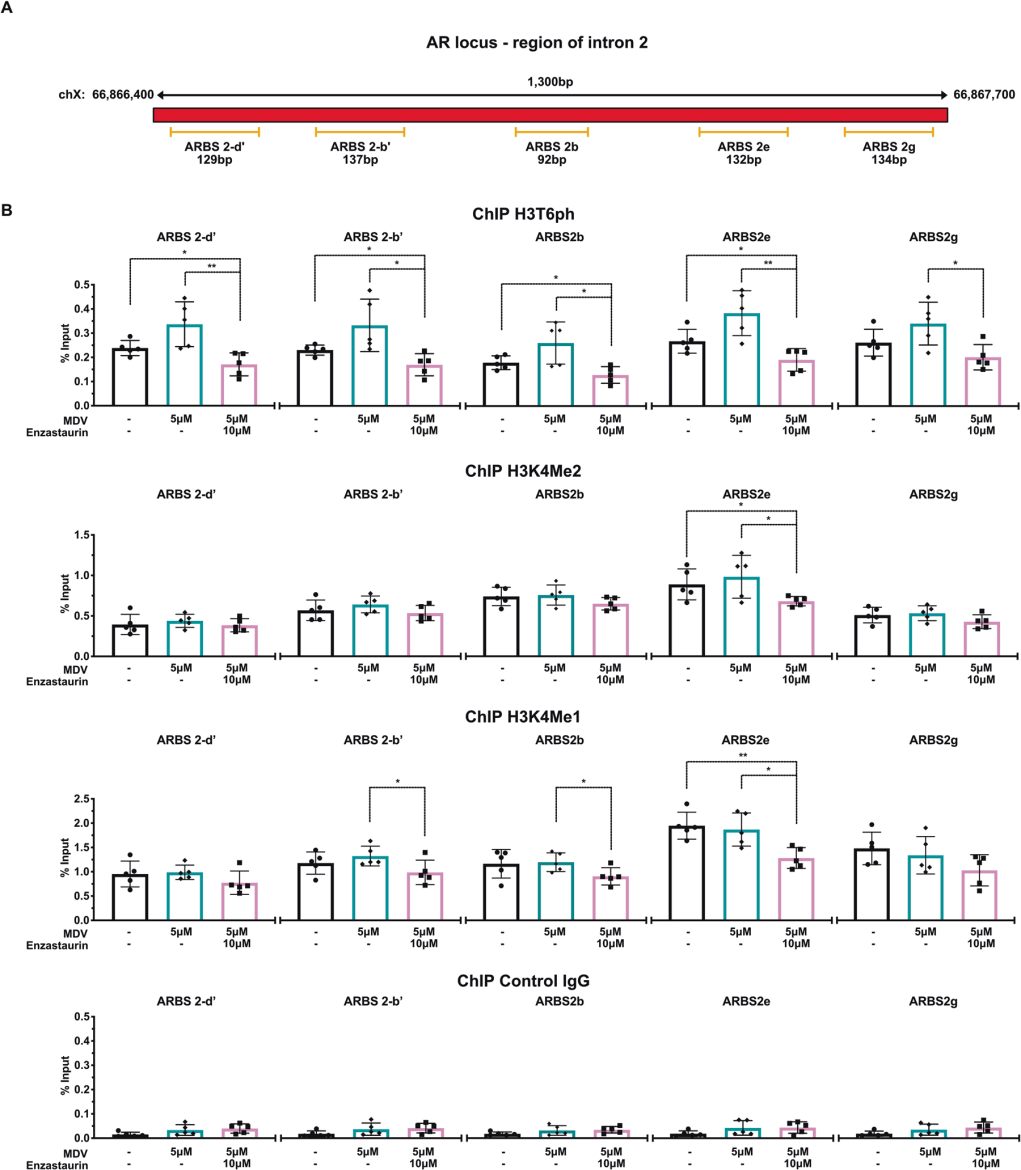
PKCβ1 inhibition reduces H3T6 phosphorylation and H3K4 methylation at the AR genomic locus. **A** Intron 2 at the AR genomic locus with primers spanning the indicated region for ChIP-qPCR analysis (Assembly GRCh37.p13). **B** VCaP cells cultured in RPMI1640 supplemented with 5% CSS for 48 h and then treated as indicated for 24 h. Samples (*N* = 5 biological replicates) processed according to the Zymo-Spin ChIP Kit with H3T6ph, H3K4Me2, H3K4Me1, and rabbit IgG antibodies. The antibody precipitated chromatin was de-crosslinked, purified, and analyzed by qRT-PCR using the primers against the regions indicated. Data is reported as percent of input and are mean ± SD (*p-value < 0.05, **p-value < 0.01).

Next, we wished to evaluate the mechanism of AR genomic locus regulation by PKCβ. We utilized a ChIP-qPCR assay with primers spaced along intron 2 of the AR genomic locus in the region previously reported to contain AR regulatory elements (Fig. 2A) [6]. Our assay reveals that enzastaurin and MDV in combination decreases histone H3T6 phosphorylation across all primer sets in this region relative to MDV alone. Further, the combination of enzastaurin and MDV reduces both histone H3K4 di- and mono-methylation at select primer sets relative to MDV alone. These observations are consistent with our proposed mechanism, where PKCβ inhibition decreases histone H3T6 phosphorylation resulting in an increase in LSD1 activity and a concomitant reduction in histone H3K4 methylation. These trends are not observed for the control IgG (Fig. 2B). Finally, we evaluated LSD1 at the AR genomic locus in our assay, and observed the presence of LSD1 in all treatment conditions and across all primer sets. Further, we generally observed a slight enrichment of LSD1 in the enzastaurin + MDV combination, and for MDV alone, when compared to vehicle. These trends were not observed for the control IgG (Supplementary Fig. S2D).

### Enzastaurin exhibits dose-dependent inhibition of AR and AR-V7 expression and suppresses rebound expression of AR-repressed genes

**Fig. 3:**
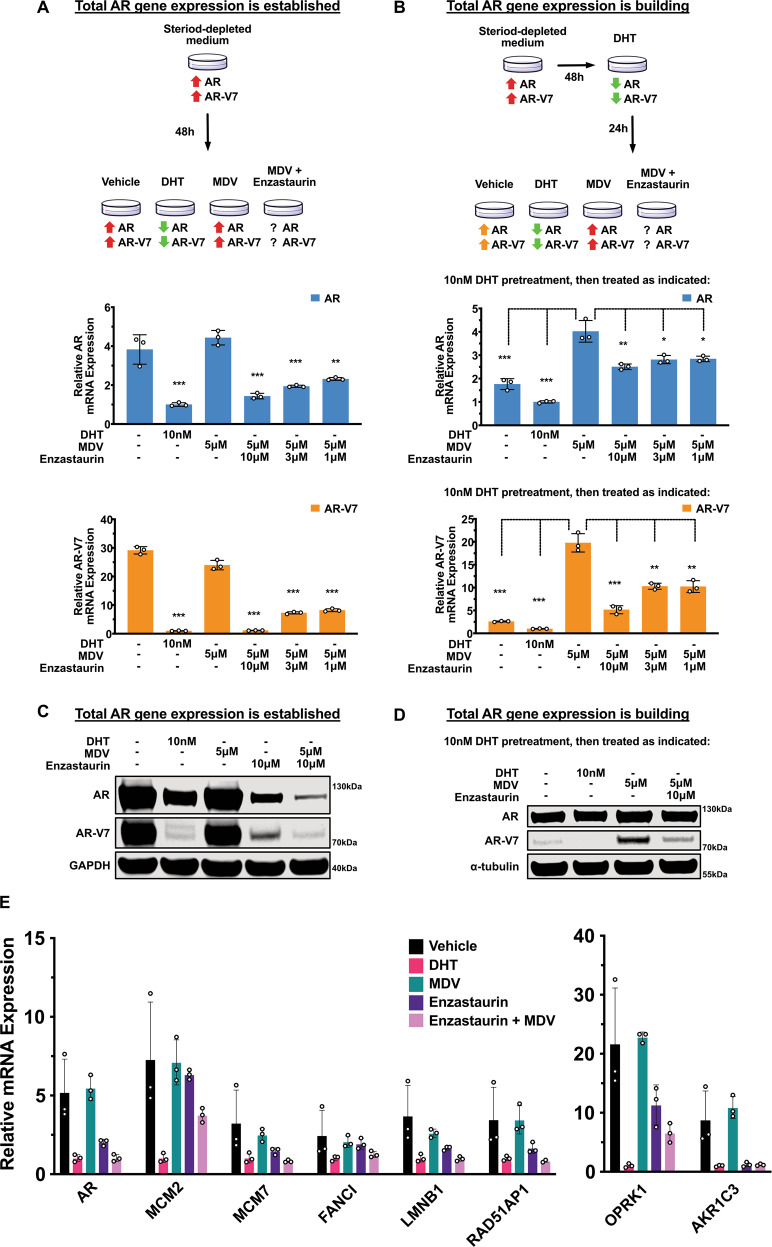
Enzastaurin effectively suppresses AR and AR-V7 mRNA and protein levels, and decreases mRNA transcripts of other androgen-dependent AR suppressed targets that are de-repressed during anti-androgen therapy. **A** VCaP cells cultured in RPMI1640 supplemented with 5% CSS for 48 h were treated in three biological replicates for 24 h as indicated and analyzed by RT-qPCR for AR and AR-V7 mRNA transcript levels. Data are mean ± SD. P-values are relative to vehicle (*p-value < 0.05; **p-value < 0.005; ***p-value < 0.0001). All mRNA expression levels are relative to GAPDH and normalized to the response for DHT. **B** VCaP cells cultured in RPMI1640 supplemented with 5% CSS for 48 h, pre-treated with DHT for 24 h, and then washed out and treated as indicated in three biological replicates for an additional 24 h and analyzed by RT-qPCR for AR and AR-V7 mRNA transcript levels. Data are mean ± SD. P-values are relative to MDV (*p-value < 0.05; **p-value < 0.005; ***p-value < 0.0001). All mRNA expression levels are relative to GAPDH and normalized to the response for DHT. **C** VCaP cells cultured in RPMI1640 supplemented with 5% CSS for 48 h were treated as indicated for 72 h and then immunoblotted for AR and AR-V7. **D** VCaP cells cultured in RPMI1640 supplemented with 5% CSS for 48 h, pre-treated with DHT for 24 h, washed out, treated as indicated for an additional 24 h, and immunoblotted for AR and AR-V7. **E** VCaP cells cultured in RPMI1640 supplemented with 5% CSS for 48 h were treated with vehicle, 10 nM DHT, 5 μM MDV, 10 μM Enzastaurin, or 5 μM MDV + 10 μM Enzastaurin in three biological replicates for 24 h and then analyzed by RT-qPCR for mRNA transcript levels of DHT-AR repressed genes. Data are mean ± SD. All mRNA expression levels are relative to GAPDH and normalized to the response for DHT.

Having established that enzastaurin represses expression of full-length AR and AR-V7, we next investigated the effect in more detail. We assessed the dose-dependent effects of enzastaurin in combination with MDV on full-length AR and AR-V7 mRNA transcript levels both when the low-androgen stress response is established, and when it is building, and observed a dose dependency for both conditions. Even at our lowest assay concentration of enzastaurin (1 μM) in combination with MDV, full-length AR mRNA transcript levels were reduced 1.7-fold and AR-V7 mRNA transcript levels were reduced 3.6-fold relative to vehicle when the low-androgen stress response is established (Fig. 3A), and full-length AR mRNA transcript levels were reduced 1.4-fold and AR-V7 mRNA transcript levels were reduced 1.9-fold relative to MDV when the low-androgen stress response is building (Fig. 3B). Further, in each condition the magnitude of the effect was greatest for AR-V7. Next, we evaluated if a reduction in AR and AR-V7 mRNA transcripts is associated with a decrease in AR and AR-V7 protein levels. We were able to observe a very clear decrease in AR and AR-V7 protein levels in the presence of our combinations (Figs. 3C, D). Enzastaurin also yielded a dose-dependent reduction in AR-V7 protein levels in the combination treatments **(**Supplementary Fig. S3A**)**. Further, when cells are treated with enzastaurin alone, a decrease in AR-V7 protein levels relative to MDV alone is observed, demonstrating that this effect is fully dependent on the PKCβ inhibitor (Fig. 3C**;** Supplementary Fig. S3B).

Next, we evaluated our combination in another AR-V7 positive cell line that is resistant to anti-androgen treatment. The 22RV1 prostate cancer cell line expresses full-length AR and AR-V7 splice variant and includes a drug-binding-resistant somatic mutation in the full-length AR LBD (H875Y). The cell line also contains a 35 kb intragenic tandem duplication of the AR gene that results in deregulation of AR splicing and contributes to AR alternative splicing and AR-V7 mRNA transcripts. This duplication event encompasses exon 3 and the neighboring sequences, including cryptic exon 3, which is found in AR-V7 [41, 42]. In 22RV1 cells, enzastaurin alone and in combination with MDV significantly decreased AR-V7 mRNA levels but not full-length AR mRNA levels, relative to vehicle and MDV. (Supplementary Fig. S3C). A reduction in AR-V7 protein levels was also observed (Supplementary Fig. S3D). We suspect the ability of enzastaurin to reduce AR-V7 in the 22RV1 cell line indicates that despite AR genomic rearrangements, transcriptional regulatory elements at the AR locus—including the role of PKCβ1—remain intact.

We hypothesized that the mechanism of AR genomic locus repression (Fig. 1A) may also be reflected at other genomic loci that are known targets of androgen-dependent repression by the full-length AR [29]. We, therefore, tested enzastaurin alone and in combination with MDV against a subset of these targets, and in almost all cases observed a reduction in mRNA transcript levels when compared to vehicle or MDV (Fig. 3E**)**. Interestingly, targets suppressed by our combination include proteins involved in genome replication (MCM2 and MCM7), in DNA damage response, and the repair of double-stranded breaks (FANCI and RAD51AP1), and metabolic enzymes involved in androgen synthesis (AKR1C3). The data indicates that combining enzastaurin with MDV can also mitigate the increases in transcription observed in response to MDV at other AR-repressed genomic loci. Finally, we evaluated expression of the AR target genes KLK2, KLK3, TMPRSS2, and ZBTB16, which demonstrate androgen-dependent activation (Supplementary Fig. S3E). Further, androgen-dependent AR activation of KLK2, KLK3, and TMPRSS2 is enhanced by PKCβ1 activity [27]. ZBTB16 activation has not been evaluated in this context. We observed that MDV inhibited transcription of all four genes, and that this inhibitory effect was further enhanced by enzastaurin.

### MDV and enzastaurin combinations demonstrate synergy in AR-V7 positive prostate cancer cells

**Fig. 4:**
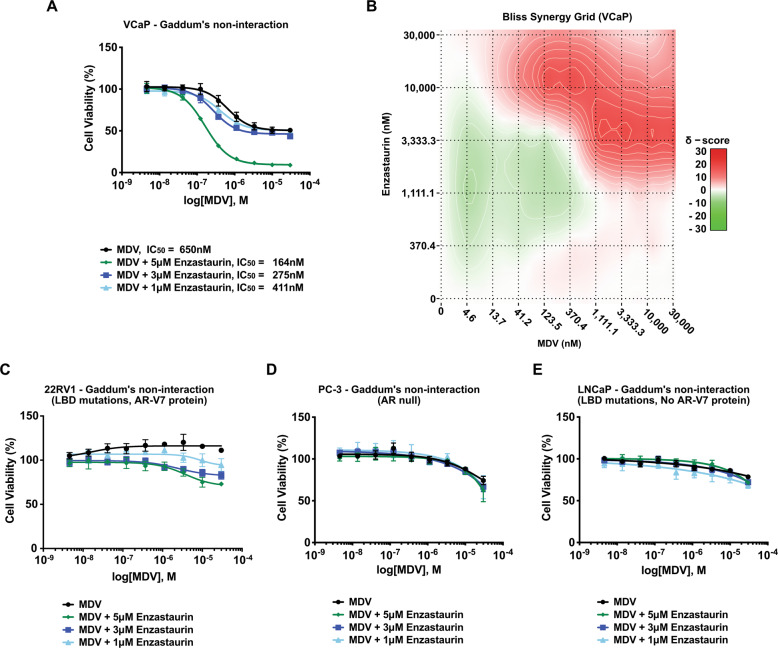
Enzastaurin and MDV combinations demonstrate synergy in the AR-V7 expressing VCaP cell line but not in other prostate cancer cell lines. **A** VCaP (5000 cells/well) cells were cultured in RPMI1640 supplemented with 5% CSS for 48 h in 96-well plates. In the presence of 0.1 nM DHT, cells were treated with a nine-point threefold dilution series of MDV (beginning at 30μM) in the presence or absence of a constant concentration of Enzastaurin for three days. Cell viability was subsequently measured in a CellTiter-Glo bioluminescence assay. Data are mean ± SD (*N* = 3 biological replicates). **B** VCaP (5000 cells/well) cells were cultured in RPMI1640 supplemented with 5% CSS for 48 h in 96 well plates. In the presence of 0.1 nM DHT, cells were treated in combination with MDV and Enzastaurin (beginning at 30 μM) in a threefold dilution series checkerboard assay for five days. Cell viability was subsequently measured in a CellTiter-Glo bioluminescence assay (*N* = 3 biological replicates) and Bliss synergy scores were calculated. **C** 22RV1 cells express AR-V7 protein and contain LBD mutation H875Y. 22RV1 (2000 cells/well) cells were cultured in RPMI1640 supplemented with 5% CSS for 48 h in 96 well plates. In the presence of 0.1 nM DHT, cells were treated with a nine-point threefold dilution series of MDV (beginning at 30 μM) in the presence or absence of a constant concentration of Enzastaurin for five days. Cell viability was subsequently measured in a CellTiter-Glo bioluminescence assay. Data are mean ± SD (*N* = 3 biological replicates). **D** PC-3 cells are AR null. PC3 (1000 cells/well) were cultured in RPMI1640 supplemented with 5% CSS for 48 h in 96 well plates. In the presence of 0.1 nM DHT, cells were treated with a nine-point threefold dilution series of MDV (beginning at 30 μM) in the presence or absence of a constant concentration of Enzastaurin for three days. Cell viability was subsequently measured in a CellTiter-Glo bioluminescence assay. Data are mean ± SD (*N* = 3 biological replicates). **E** LNCaP cells do not have AR-V7 protein and contain LBD mutation T878A. LNCaP (5000 cells/well) cells were cultured in RPMI1640 supplemented with 5% CSS for 48 h in 96 well plates. In the presence of 0.1 nM DHT, cells were treated with a nine-point threefold dilution series of MDV (beginning at 30 μM) in the presence or absence of a constant concentration of Enzastaurin for three days. Cell viability was subsequently measured in a CellTiter-Glo bioluminescence assay. Data are mean ± SD (*N* = 3 biological replicates).

To test the hypothesis that reducing full-length AR and AR-V7 splice variants during androgen blockade will increase anti-androgen effectiveness in our VCaP cell line, we assessed our combinations in several drug synergy assays. First, we assessed synergy by Gaddum’s non-interaction, also known as the Highest Single Agent model. In this model, a synergistic combination will yield a greater effect than a single agent alone at the same concentrations [43, 44]. We, therefore, determined the IC_50_ values on VCaP cell viability for serial dilutions of MDV alone and at constant concentrations of enzastaurin. As expected, enzastaurin improved the effectiveness of MDV when measured by IC_50_ (Fig. 4A). Second, we assessed the synergy of our combination in a checkerboard assay using the SynergyFinder web application and a Bliss Independence model to generate a map of synergistic (red) and antagonistic (green) interactions (Fig. 4B) [45]. We again observed synergy, with the most robust combinations occurring when the concentrations of MDV and enzastaurin are both above 1 μM. Third, we used CompuSyn 1.0 to calculate Chou-Talalay combination indices (CIs) at different fractional inhibitions (Fa) for a dilution series of our combination in a 1:1 ratio [46, 47]. The CIs reveal synergy across our dilution series, and a sampling of the CIs is presented in Supplementary Table S2.

Next, we assessed our combination by Gaddum’s non-interaction in 22RV1 prostate cancer cells. The 22RV1 cells display uninhibited growth in the presence of MDV despite the antagonism of full-length AR androgen-dependent activation, however, knockdown of AR-V7 has been shown to sensitize this cell line to MDV [48]. In our assay, only prolonged treatment of MDV in combination with enzastaurin reveals an inhibitory effect, but could not be fit to a nonlinear regression model **(**Fig. 4C**)**. Importantly, our data indicate that repression of AR-V7 by enzastaurin does allow for MDV-dependent growth inhibition. Overall, we show that our combination improves the response of AR-V7 positive prostate cancer cells to MDV.

Finally, we assessed our combination by Gaddum’s non-interaction in the PC-3 and LNCaP prostate cancer cell lines. The PC-3 cell line is AR-null, and as expected, neither MDV nor our combination of MDV with enzastaurin showed an effect on cell viability (Fig. 4D). The LNCaP cell line contains full-length AR protein with a T878A somatic mutation in its LBD and is resistant to anti-androgens [49, 50]. LNCaP cells do not express AR-V7 protein, but AR-V7 splice variant mRNA can be detected [5] (Supplementary Fig. S4A and Supplementary Fig. S4B). We observed a weak response for MDV alone and with our combinations in LNCaP cells (Fig. 4E and Supplementary Figure S4C). This response is the most pronounced at higher concentrations of MDV (Supplementary Figure S4C), however, the response achieved in our dose series is not sufficient for the calculation of an IC_50_ value. We suspect this to be due to the resistant nature of the cell line.

### Enzastaurin is compatible with next-generation AR degrader strategies in AR-V7 positive prostate cancer cells

**Fig. 5:**
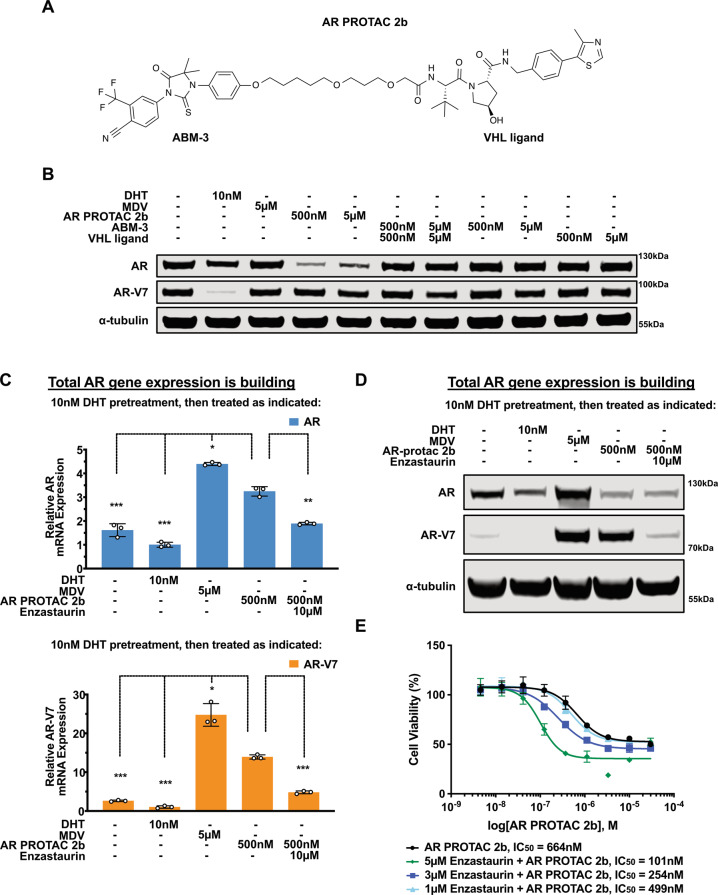
Enzastaurin is compatible with the next-generation AR degraders. **A** An AR PROTAC consisting of ABM-3 and the VHL ligand connected by a linker. ABM-3 structurally resembles the anti-androgen MDV. **B** VCaP cells cultured in RPMI1640 supplemented with 5% CSS for 48 h were treated as indicated for 24 h and then immunoblotted for AR and AR-V7. **C** VCaP cells cultured in RPMI1640 supplemented with 5% CSS for 48 h, pre-treated with DHT for 24 h, and then washed out and treated as indicated in three biological replicates for an additional 24 h and analyzed by RT-qPCR for AR and AR-V7 mRNA transcript levels. Data are mean ± SD. P-values are relative to **AR PROTAC 2b** (*p-value < 0.05; **p-value < 0.005; ***p-value < 0.0001). All mRNA expression levels are relative to GAPDH and normalized to the response for DHT. **D** VCaP cells cultured in RPMI1640 supplemented with 5% CSS for 48 h, pre-treated with DHT for 24 h, and then washed out and treated as indicated for an additional 72 h and then immunoblotted for AR and AR-V7. **E** VCaP (5000 cells/well) cells were cultured in RPMI1640 supplemented with 5% CSS for 48 h in 96 well plates. In the presence of 0.1 nM DHT, cells were treated with a nine-point threefold dilution series of **AR PROTAC 2b** (beginning at 30 μM) in the presence or absence of a constant concentration of Enzastaurin for seven days. Cell viability was subsequently measured in a CellTiter-Glo bioluminescence assay. Data are mean ± SD (*N* = 3 biological replicates).

The next generation of AR antagonists, the AR degraders, are in development and under evaluation in clinical trials. These bivalent molecules consist of an anti-androgen tethered to a ligand that recruits an E3 ligase to the full-length AR, causing it to be ubiquitinated and targeted for degradation [51–53]. We hypothesized that these molecules will not degrade the AR-V7 splice variant due to the deletion of the LBD. Additionally, degradation of full-length AR protein will ablate AR-dependent gene repression and allow transcriptional activation of the AR genomic locus, thus increasing total AR mRNA transcript levels and potentially driving resistance through AR-V7 **(**Fig. 1A**(middle))**. We, therefore, wished to determine if our combination strategy with enzastaurin could effectively reduce AR-V7 splice variant mRNA transcripts in the presence of an AR degrader. First, we synthesized a biologically evaluated AR degrader **(AR PROTAC 2b)** reported previously (Fig. 5A) [53]. This degrader consists of the anti-androgen ABM-3, which structurally resembles MDV, tethered to a ligand that recruits the von Hippel-Lindau (VHL) E3 ligase to the full-length AR causing degradation [54]. We then evaluated **AR PROTAC 2b**, as well as its separated components, in VCaP cells to confirm that the full-length AR protein is degraded by the bivalent molecule, but the AR-V7 splice variant protein persists. The bivalent molecule proved highly effective at 500 nM. A slight hook effect for full-length AR degradation was observed at 5 μM, which is an expected effect for bivalent degrader molecules (Fig. 5B) [55].

Having confirmed its efficacy against full-length AR, we next evaluated **AR PROTAC 2b** in several of our established assays. First, we assessed the VCaP cell line after DHT wash out with **AR PROTAC 2b**, an assay in which MDV alleviates AR genomic locus suppression resulting in AR rebound expression. In this experiment, we observed rebound expression of AR and AR-V7 after treatment with **AR PROTAC 2b**, although the expression was less than that observed with MDV (Fig. 5C). Enzastaurin in combination with **AR PROTAC 2b** suppressed AR locus expression and reduced the level of AR and AR-V7 mRNA transcripts by 1.7-fold and 2.9-fold respectively, relative to **AR PROTAC 2b** alone. The effect was larger when the combination of enzastaurin and **AR PROTAC 2b** was compared to MDV, where the relative levels of full-length AR and AR-V7 mRNA transcripts were reduced by 2.3-fold and 5.1-fold respectively. We also evaluated AR-V7 protein levels after the same treatments. AR-V7 protein levels when **AR PROTAC 2b** and enzastaurin are combined were remarkably lower than **AR PROTAC 2b** alone (Fig. 5D). Finally, we assessed **AR PROTAC 2b** in combination with enzastaurin in Gaddum’s non-interaction assay, revealing that combination with enzastaurin improves the effectiveness of **AR PROTAC 2b** (Fig. 5E).

### MDV and enzastaurin in combination demonstrate greater efficacy than MDV alone in vivo against VCaP xenografts

**Fig. 6:**
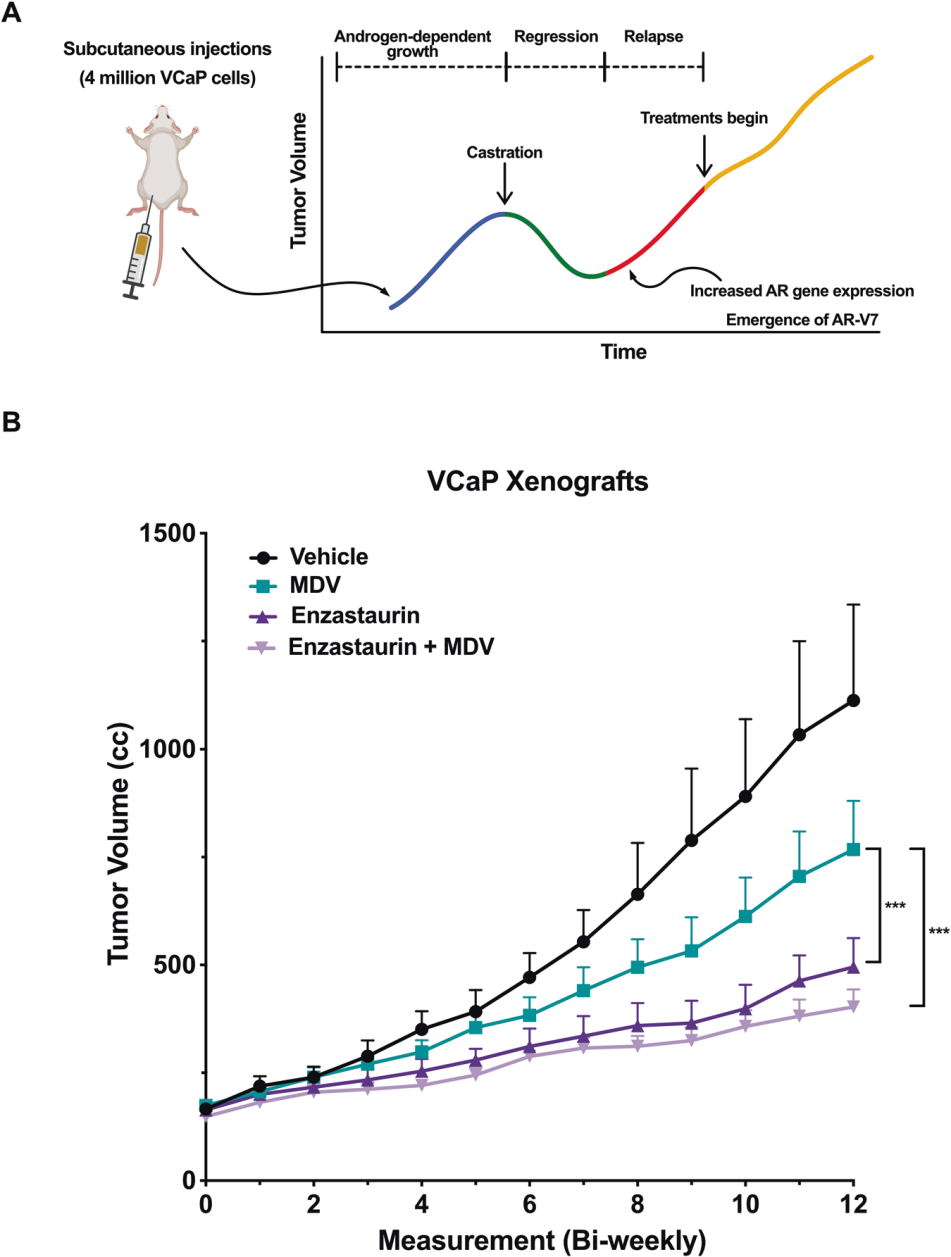
VCaP xenografts respond positively to Enzastaurin and MDV in combination. **A** VCaP cells were introduced by subcutaneous flank injection. The xenografts initially developed in mice with circulating androgen. Once tumor volumes reached ~100 cc, mice were castrated and the xenografts subsequently shrank until androgen independence was acquired. Xenografts then relapsed and treatments began when tumors reached ~150 cc. **B** Tumor volumes in mice bearing VCaP (AR-V7 positive) xenografts (*N* = 8-9 mice per arm). Measurements were recorded biweekly for a total of six weeks. Treatments consisted of vehicle, MDV (10 mg/kg), Enzastaurin (50 mg/kg BID), Enzastaurin (50 mg/kg BID) + MDV (10 mg/kg) (***P-value < 0.0001). Data are mean ± SEM.

We designed an in vivo study using VCaP xenografts to mimic reactivation of androgen receptor signaling in CRPC to validate the effectiveness of our combination relative to the MDV monotherapy [56]. Once the VCaP xenografts were established in castrated male mice (Fig. 6A), dosing followed a schedule of five days on and two days off for a total of six weeks with bi-weekly tumor volume measurements. Dosing for the monotherapies was determined from literature precedent [57–59] and dosing for the combination therapy was determined in a tolerability study (Supplementary Fig. S5A). The combination therapy proved more effective than the MDV monotherapy demonstrating that enzastaurin can augment the effect of MDV in vivo (Fig. 6B). Not surprisingly, the enzastaurin monotherapy also proved more effective than the MDV monotherapy due to its ability to suppress total AR gene expression. Upon completion of the study, we analyzed the VCaP xenografts by immunoblot. Our analysis reveals an average reduction in both AR-V7 and histone H3T6 phosphorylation in the combination therapy and enzastaurin monotherapy relative to the MDV monotherapy (Supplementary Fig. S5B, Supplementary Table S3).

## Discussion

CRPC is an aggressive cancer that follows relapse of hormone-naïve prostate cancer. Prognosis is particularly poor when AR-V7 is detected, as ARSi offers little benefit; and patients experience shorter PSA progression-free survival and lower overall survival than patients negative for AR-V7 [3]. The AR-V7 splice variant protein lacks its LBD, and largely consists of an unstructured N-terminal domain and a DNA-binding domain that is highly conserved across the nuclear receptor superfamily [60]. This complicates direct inhibition by a small molecule due to the lack of a ‘druggable’ pocket that can be targeted with high specificity and selectivity.

Prior work has indicated that the full-length AR auto-regulates its own genomic locus in response to androgen. In a high-androgen environment the full-length AR localizes to intron 2 of the AR genomic locus and recruits LSD1 to remove methyl marks from histone H3K4 to suppress transcriptional activity at the locus [6, 31]. However in a low-androgen environment, AR genomic locus repression is alleviated [6], and transcription at the locus increases [5, 6, 9]. PKCβ1 is reported to facilitate a subset of transcriptional programs by phosphorylating histone H3T6 to block lysine demethylase activity, thus allowing H3K4 methylation to persist [27]. We hypothesized that in the absence of androgen-bound full-length AR, PKCβ1 is active at the AR genomic locus and promoting transcription. Since spliceosome assembly and pre-mRNA processing occurs co-transcriptionally and is dependent on chromatin modifications, we proposed that by employing an epigenetic strategy focused on PKCβ1 inhibition we could target total AR expression, including AR-V7. We hypothesized that this approach would reduce both full-length AR and the undruggable AR-V7 transcription factor, providing an opportunity to antagonize AR-V7-driven prostate cancer growth. Further, we anticipated that this approach would be amenable to combination with current anti-androgen therapies, allowing co-targeting of AR-V7 and full-length AR.

We evaluated the clinical PKCβ inhibitor enzastaurin for its ability to suppress both full-length AR and AR-V7 mRNA transcripts according to our proposed mechanism. Our results indicate that enzastaurin is able to reduce total AR mRNA transcripts, including AR-V7, in the presence of MDV. In particular, we also observed that reductions in AR-V7 mRNA transcripts resulted in a marked reduction in AR-V7 protein levels. Notably, the magnitude of this effect is greater for AR-V7 than it is for full-length AR at both the mRNA and protein levels. We evaluated the kinases targeted by enzastaurin and determined that its effectiveness is dependent on PKCβ inhibition. Furthermore, the data indicates that GSK3α and GSK3β inhibition are responsible for a partial reduction in AR-V7 mRNA transcript levels. A number of transcription factors are direct substrates of the GSK3 kinases, and we speculate that the partial effect of GSK3 inhibition may be due to this regulation [61, 62]. Further, reducing GSK3 activity is reported to sensitize PKCβ to small molecule inhibitors, therefore another potential mechanism is that co-targeting of GSK3 and PKCβ by enzastaurin improves the effect of PKCβ inhibition [63]. We concluded that the effectiveness of enzastaurin is mediated by inhibition of PKCβ1 and likely enhanced by inhibition of GSK3α and GSK3β through an additional mechanism.

We used several different synergy models to demonstrate that enzastaurin synergizes with MDV to inhibit AR-V7 positive prostate cancer cell growth. The combination exhibited a pronounced effect for cell growth inhibition in AR-V7 positive prostate cancer cell lines. Importantly, no effect was observed for our combinations in other prostate cancer model cell lines that do not express AR-V7. Interestingly, enzastaurin also proved efficacious for reducing AR-V7 splice variant levels in combination with an AR degrader and improved the effectiveness of the degrader. We assessed the effectiveness of our enzastaurin and MDV combination against the MDV monotherapy in a VCaP xenograft mouse model and found that the combination was more effective than MDV alone.

In this work we have attempted to offer a mechanistic explanation for regulation of AR-V7 splicing by our combination therapy. However, protein kinase C regulates a complex signaling network and we recognize that the splicing effects we observe could in part be indirectly regulated through other PKC substrates in addition to our proposed mechanism. Finally, this work was primarily performed in the VCaP cell line, which contains wild-type full-length AR and demonstrates a unique responsiveness to both DHT and MDV compared to other prostate cancer cell lines [5, 6]. Our combination therapy demonstrated the greatest effect in the VCaP cell line indicating that it may not be as effective in other settings.

To date, enzastaurin has been evaluated in two Phase-II clinical trials against metastatic CRPC [64, 65]. In the first trial, patients were grouped into two cohorts: those with progressive non-metastatic disease and those with progressive metastatic disease following treatment with docetaxel-based chemotherapy. Both cohorts were provided enzastaurin monotherapy. A mild response was observed for patients with progressive metastatic disease [65]. In the second trial, patients with metastatic CRPC received docetaxel with prednisone, with or without enzastaurin. No significant difference was observed between the two groups [64]. At the time of these trials, enzalutamide and other AR antagonists were not considered standard of care therapies, but today, ARSi therapies are far more prevalent and are correlated with a significant increase in the frequency of AR-V7 positive metastatic CRPC [18, 66]. Further, assays are now clinically available for the detection of AR-V7 positive circulating tumor cells and AR-V7 levels are dictating patient selection for clinical trials [18, 66]. While the activity of enzastaurin was modest in the pre-ARSi era, due to the establishment of ARSi therapies and the prevalence of AR-V7-positive metastatic CRPC, we feel that our in vitro and in vivo work justifies PKCβ1 inhibition in combination with AR antagonists as a viable strategy for further clinical evaluation against AR-V7-positive prostate cancer in an AR-V7 biomarker-selected trial. In conclusion, we report a new mechanistic approach based on reducing full-length AR and AR-V7 splice variant protein levels that increases the sensitivity of AR-V7 prostate cancer cells to AR antagonism.

## Methods

### Cell culture and reagents

All cell lines in this study (VCaP, CRL-2876; PC-3, CRL-1435; LNCaP, CRL-1740) were obtained from the American Type Culture Collection (ATCC), with the exception of the 22RV1 cell line, which was provided to us by Felix Feng. VCaP, PC-3 and LNCaP cells were cultured in 5% CO_2_ at 37 °C with Dulbecco’s modified Eagle’s medium (DMEM) (ATCC, 30-2002) supplemented with 10% (v/v) heat-inactivated fetal bovine serum (Axenia BioLogix). 22RV1 cells were cultured in 5% CO_2_ at 37 °C with Roswell Park Memorial Institute medium (RPMI) (ATCC, 30-2001) supplemented with 10% (v/v) heat-inactivated fetal bovine serum (Axenia BioLogix). Cellular assays were performed in RPMI medium supplemented with 5% (v/v) charcoal stripped fetal bovine serum (CSS) (Gibco, A33821). Cells were periodically tested for contamination using the MycoAlert Plus Mycoplasma Detection Kit (Lonza). Dihydrotestosterone was purchased from Sigma Aldrich. Enzalutamide (MDV) was purchased from MedChem Express. Enzastaurin was purchased from both Selleck Chemicals and MedChem Express. All reagents used for synthesis of the AR degrader were obtained from Sigma Aldrich, Acros Organics, Cayman Chemicals or AstaTech. The AR degrader was synthesized as described [53].

### SDS-PAGE and Immunoblotting

Treated cells (~500,000 – 1,000,000 cells/well) were lysed with RIPA buffer or with 20 mM Tris-HCl, pH 7.5, 0.5 mM EDTA, 0.5 mM EGTA, 1 mM DTT, 10% Glycerol and 0.5% IGEPAL CA-630 supplemented with phosphate inhibitors (Roche, PhosSTOP) and protease inhibitors (Roche, cOmplete Protease Inhibitor Cocktail Tablets), and protein concentration was determined by either a Bradford Assay (Bio-Rad, Protein Assay Dye Reagent Concentrate) or a bicinchoninic acid assay (Thermo Fisher Scientific, Pierce BCA Protein Assay Kit). Protein lysates were resolved by SDS-PAGE, transferred to nitrocellulose membranes (Bio-Rad) and blocked using either 5% milk or 5% BSA in TBST buffer (1X Tris-buffered saline (TBS), 0.1% Tween-20). Nitrocellulose membranes were immunoblotted with antibodies against AR (1:1000 in 5% milk/TBST; Santa Cruz Biotech, sc-7305), AR-V7 (1:500 5% milk/TBST; Precision Antibody, AG-10008); PKCβ1 (1:500 in 5% Milk/TBST; Abcam, ab195039), H3T6ph antibody (1:500 in 5% BSA/TBST; Abcam, ab222768), Histone H3 (1:2000 5% BSA/TBST; Cell Signaling Technology, 4499), α-tubulin (1:1000 in 5% BSA/TBST; Cell Signaling Technology, 3873) and GAPDH (1:1000 in 5% BSA/TBST; Proteintech, 60004-1-lg). Following the primary antibodies, nitrocellulose membranes were incubated with IRDye secondary antibodies (LI-COR Biosciences) and analyzed on an Odyssey Imaging System (LI-COR Biosciences) according to manufacturer instructions.

### qRT-PCR analysis

RNA from cells (~500,000 cells/well) treated in biological replicates (*N* = 3) were isolated using the RNeasy Plus Mini Kit (QIAGEN) according to manufacturer instructions. Reverse transcription was performed using the SuperScript III First-Strand Synthesis SuperMix for qRT-PCR (Life Technologies) according to manufacturer instructions. The reverse transcription products were evaluated by qRT-PCR using the Maxima SYBR Green qPCR Master Mix (Life Technologies) on a Bio-Rad CFX Touch Real-Time PCR system according to manufacturer instructions. GAPDH served as a reference gene. All samples were evaluated using the ΔΔCq method under the gene expression tab in the Bio-Rad CFX Maestro for Mac 1.1 software. Primer sequences are as follows: AR forward: 5’-TCT TGT CGT CTT CGG AAA TGT-3’, AR reverse: 5’-AAG CCT CTC CTT CCT CCT GTA-3’; AR-V7 forward: 5’-CAG GGA TGA CTC TGG GAG AA-3’, AR-V7 reverse: 5’-GCC CTC TAG AGC CCT CAT TT-3’; GAPDH forward: 5’-GGA CCT GAC CTG CCG TCT AG AA-3’, GAPDH reverse: 5’-GGT GTC GCT GTT GAA GTC AGA G-3’; MCM2 forward: 5’-ATT TCG TCC TGG GTC CTT TC-3’, MCM2 reverse: 5’-GCT GGT AGT TCT GAT AGA TGG T-3’; MCM7 forward: 5’-GGA TGC CAC CTA TAC TTC TGC-3’, MCM7 reverse: 5’-CCT TTG ACA TCT CCA TTA GCC T-3’; FANCI forward: 5’-CAA TGA GGA ACA GAG TGG TGA-3’, FANCI reverse: 5’-GCC TAG TTC ATA GTC CAA TTT GAT G-3’; LMNB1 forward: 5’-GGA AAT CAG TGC TTA CAG GAA AC-3’, LMNB1 reverse: 5’-CTT GAG GAT GCT CGG GAT AC-3’; RAD51AP1 forward: 5’-GTC TTC AGA TAC CAC TAG GAA ACC-3’; RAD51AP1 reverse: 5’-CTG CTG CTA CTT CTG CTA CC-3’; OPRK1 forward: 5’-TCA TCA ATA TCT GCA TCT GGC T-3’; OPRK1 reverse: 5’-AAG GAG CAC TCA ATG ACA TCG-3’; AKR1C3 forward: 5’-GGC CAC TTC ATG CCT GTA-3’, AKR1C3 reverse: 5’-GAA CCC AGC TTC TAT TGC TAA-3’.

### Multiplexed inhibitor beads (MIB) assay

Kinase chromatography, mass spectrometry and analytical processing were performed as described previously [40]. Briefly, cells growing in RPMI 1640 (ATCC, 30-2001) supplemented with 5% (v/v) charcoal-stripped fetal bovine serum (CSS) (Gibco, A33821) for 48 h were treated in three biological replicates for 24 h with DMSO, DHT, or MDV and then collected in PBS. Samples were lysed in 150 mM NaCl buffer with protease and phosphatase inhibitors, and then diluted in 1 M NaCl binding buffer. Affinity purification was performed with gravity chromatography after pre-clearing. The bound kinases were washed and eluted followed by extraction/precipitation, tryptic digest, and desalting. Liquid chromatography-tandem mass spectrometry (LC/MS-MS) was performed on a Q-Exactive with in-line high-performance liquid chromatography (HPLC) at the Thermo Fisher Scientific Proteomics Facility for Disease Target Discovery at UCSF and the J. David Gladstone Institutes. Peptide identification was done with MaxQuant, label-free quantification with Skyline [67], and statistical analysis with MSstat [68].

### ChIP qRT-PCR assay

Cells growing in 10 or 15-cm plates were cultured in RPMI 1640 (ATCC, 30-2001) supplemented with 5% (v/v) charcoal stripped fetal bovine serum (CSS) (Gibco, A33821) for 48 h. Plates were then treated in biological replicates with vehicle, 5 μM MDV or 5 μM MDV + 10 μM Enzastaurin for 24 h. Samples were subsequently processed using the Zymo-Spin ChIP Kit (D5209) and either a H3T6ph antibody (Abcam, ab222768), H3K4Me2 (Cell Signaling Technology, 9725), H3K4Me1 (Cell Signaling Technology, 5326), LSD1 (Abcam, 129195) or a rabbit IgG antibody (Cell Signaling Technology, 2729). The precipitated DNA was evaluated by qRT-PCR using the Maxima SYBR Green qPCR Master Mix (Life Technologies) on a Bio-Rad CFX Touch Real-Time PCR system according to manufacturer instructions. Data is reported as percent of input. Primer sequences are as follows: ARBS2d’ forward: GCT CAG AGA GGT TTT AGT TGT G, ARBS2d’ reverse: CAA AAT GTC TAA GCT GGA AGC AC; ARBS2b’ forward: GTC TTG CTT TCC TAG AAG GTG AC; ARBS2b’ reverse: CAA GGA GAA AAT CTG AGT CCT GAG; ARBS2b forward: CAC ATG GAG TGC TGT TTG GT, ARBS2b reverse: GTA AAC ATC AGT GAG GAT GGT G; ARBS2e forward: GCA GAG AGT TTT TGG TGC ATA TC, ARBS2e reverse: CAA AGA TAC CTG ATG AAG GCT CTG; ARBS2g forward: CAG ACT TTA GAT TTA GGG GTT GG, ARBS2g reverse: GTC TAT GGC TGC TTT CAT CCT AC.

### Drug synergy assays

Cells were seeded into white 96-well clear flat bottom plates (Corning, 3903) in RPMI 1640 (ATCC, 30-2001) supplemented with 5% (v/v) charcoal stripped fetal bovine serum (CSS) (Gibco, A33821) for 48 h. Cells were then treated in biological replicates (*N* = 3) accordingly: Gaddum’s non-interaction: In the presence of 0.1 nM DHT, MDV in a nine-point threefold dilution series at Enzastaurin concentrations of 5 μM, 3 μM, 1 μM or 0 μM; Checkerboard assay: In the presence of 0.1 nM DHT, MDV and Enzastaurin checkerboarded in a threefold dilution series; Chou-Talalay combination indices: In the presence of 0.1 nM DHT, MDV and Enzastaurin as monotherapies or as a 1:1 concentration ratio combination in a nine-point threefold dilution series. Cell viability was assessed after three or five days using a CellTiter-Glo luminescence-based assay (Promega). The CellTiter-Glo reagent was diluted fivefold in PBS and added to cells in a 1:1 ratio with the cellular growth medium. Plates were incubated with shaking at room temperature for 20 min and then the luminescence signal was recorded on a Tecan Spark plate reader. Bliss synergy scores were calculated using https://synergyfinder.fimm.fi/. Chou-Talalay combination indices were determined using CompuSyn 1.0 (negative viability measurements were substituted with a value of 0.0001).

### VCaP xenograft study and preparation for Immunoblotting

All mouse manipulations were performed in accordance with the University of California, San Francisco’s Institutional Animal Care and Use Committee. All animals were housed in specific pathogen-free conditions and cared for according to the International Association for Assessment and Accreditation of Laboratory Animal Care policies and certification (IACUC protocol ANI179937). All surgeries were performed under isoflurane anesthesia. Six- to eight-week-old male NSG mice (005557, Jackson Lab, Bar Harbor, ME) were bred in house and housed with ad libitum food and water on a 12 h light cycle at the UCSF Preclinical Therapeutics Core vivarium. VCaP xenografts were introduced to the right flanks of mice by subcutaneous injection (4 million cells in 100 μL; 1:1 ratio of Corning Matrigel and serum-free DMEM). Mice were castrated on a rolling basis as the VCaP xenografts reached sizes of ~100 mm^3^. The xenografts would shrink following castration, and then regrow. Mice were enrolled evenly and randomly into each arm of the study on a rolling basis as the xenografts approached sizes of ~150 mm^3^. Mice were dosed by oral gavage on a schedule five days on followed by two days off. Arms consisted of: Vehicle (1% carboxymethyl cellulose, 0.1% Tween-80, 5% DMSO), MDV – 10 mg/kg (1% carboxymethyl cellulose, 0.1% Tween-80, 5% DMSO), Enzastaurin – 50 mg/kg BID (5% DMSO, 15% Captisol) and MDV + Enzastaurin. Tumor volumes and body weights were collected biweekly over the course of the six week study. Tumor volumes were assessed by 2D caliper measurements and volume was calculated according to the volume of an ellipsoid (*V* = 0.52 x (width)^2^ x length). The xenografts were collected and flash frozen upon termination of the time course study. Pieces of the xenografts were crushed under liquid nitrogen, and the tissue was lysed with RIPA buffer containing 1X PhosSTOP, 1X PIC and 1 mM PMSF on ice with occasional vortexing. Samples were analyzed according the protocol outlined in the SDS Page and Immunoblotting section.

### Statistical analysis

qRT-PCR ΔΔCt values were calculated using the Bio-Rad CFX Maestro software and then plotted using Graphpad Prism 8 as the mean ± SD with individual data points shown, and the Tukey P-values are reported from the ANOVA tab. Immunoblots were processed with Image Studio Lite 5.2.5 (LI-COR). Gaddum’s non-interaction data is represented as mean ± SD, and IC_50_ values were determined in Graphpad Prism 8 using a log(inhibitor) vs response – variable slope (four parameter) model. The Bliss synergy grid was modeled using https://synergyfinder.fimm.fi/. Chou-Talalay combination indices were calculated using CompuSyn 1.0. P-values for ChIP-qPCR assay calculated in Microsoft Excel using the two-tail Student’s T-test function assuming equal variance. P-values for the xenograft studies were calculated by two-way ANOVA in Graphpad Prism 8.

## Supplementary information

Supplementary Tables

Supplementary Figures

## Data Availability

The full immunoblot images are provided in Supplementary Fig. S6. All data generated or analyzed during the current study are included in this published article.
