# Supplementary material for: Targeting a splicing-mediated drug resistance mechanism in prostate cancer by inhibiting transcriptional regulation by PKCβ1
Source: Oncogene. 2022 Jan 27;41(11):1536–49. doi: 10.1038/s41388-022-02179-z (PMC8913362; doi:10.1038/s41388-022-02179-z)
Supplement: Supplementary file 1 — Supplementary Tables [file 41388_2022_2179_MOESM1_ESM.docx]

**Targeting a Splicing-mediated Drug Resistance Mechanism in Prostate Cancer by Inhibiting Transcriptional Regulation by PKCβ**

James E. Melnyk^1^, Veronica Steri^2,3^, Hao G. Nguyen^2,4^, Y. Christina Hwang^2,5^, John D. Gordan^2,5^, Byron Hann^2,3^, Felix Y. Feng^2,4,6,7^, Kevan M. Shokat^1,8^

^1^Department of Cellular and Molecular Pharmacology, University of California, San Francisco, San Francisco, CA 94158, USA

^2^Helen Diller Family Comprehensive Cancer Center, University of California, San Francisco, San Francisco, CA 94158, USA

^3^Preclinical Therapeutics Core, University of California San Francisco, San Francisco, CA 94158, USA

^4^Department of Urology, University of California, San Francisco, San Francisco CA 94143, USA

^5^Department of Medicine and Division of Hematology/Oncology, University of California, San Francisco, San Francisco, CA 94158, USA

^6^Department of Radiation Oncology, University of California, San Francisco, San Francisco, CA 94143, USA

^7^Department of Medicine, University of California, San Francisco, San Francisco, CA 94143, USA

^8^Howard Hughes Medical Institute, University of California, San Francisco, San Francisco, CA 94143, USA.

**Supplementary Tables and Legends**

| **Lane** | **1** | **2** | **3** | **4** | **5** | **6** |
| --- | --- | --- | --- | --- | --- | --- |
| Normalized AR | 1.000 | 0.699 | 0.724 | 0.816 | 1.138 | 1.127 |
| Normalized AR-V7 | 1.000 | 0.533 | 0.493 | 0.704 | 1.196 | 1.223 |

**Supplementary Table S1.** Quantification of immunoblots for Figure S1A relative to α-tubulin and normalized to lane 1.

**Chou-Talalay Combination Index (CI)**

**(VCaP)**

| **Fractional Inhibition (Fa)** | **CI** |
| --- | --- |
| 0.1 | 0.27315 |
| 0.3 | 0.24979 |
| 0.5 | 0.23617 |
| 0.7 | 0.2233 |
| 0.9 | 0.20422 |

**Supplementary Table S2. Combination indices (CIs) derived from drug synergism in the VCaP cell line.** VCaP (5000 cells/well) cells cultured in RPMI1640 supplemented with 5% CSS for 48h in 96 well plates. In the presence of 0.1nM DHT, cells were treated in a nine-point threefold dilution series with MDV, Enzastaurin, or MDV and Enzastaurin in combination in a 1:1 concentration ratio (dilution series from 4.57 nM to 30 μM). Cell viability measured in a CellTiter-Glo bioluminescence assay after five days. CI values were calculated using CompuSyn 1.0 (N = 3 biological replicates). CI < 1 indicates synergism, CI = 1 indicates additivity, and CI > 1 indicates antagonism.

|  | Vehicle | MDV | Enzastaurin | Enzastaurin + MDV |
| --- | --- | --- | --- | --- |
| Average AR-V7 | 0.61 ± 0.30 | 0.61 ± 0.15 | 0.50 ± 0.11 | 0.48 ± 0.14 |
| Average Phospho-H3T6 | 1.77 ± 0.68 | 2.25 ± 0.41 | 1.72 ± 0.48 | 1.63 ± 0.53 |

**Supplementary Table S3.** Quantification averages for AR-V7 and Phospho-H3T6 for Supplementary Figure S5 immunoblot.
