# Supplementary material for: Targeting a splicing-mediated drug resistance mechanism in prostate cancer by inhibiting transcriptional regulation by PKCβ1
Source: Oncogene. 2022 Jan 27;41(11):1536–49. doi: 10.1038/s41388-022-02179-z (PMC8913362; doi:10.1038/s41388-022-02179-z)
Supplement: Supplementary file 2 — Supplementary Figures [file 41388_2022_2179_MOESM2_ESM.docx]

**Targeting a Splicing-mediated Drug Resistance Mechanism in Prostate Cancer by Inhibiting Transcriptional Regulation by PKCβ**

James E. Melnyk^1^, Veronica Steri^2,3^, Hao G. Nguyen^2,4^, Y. Christina Hwang^2,5^, John D. Gordan^2,5^, Byron Hann^2,3^, Felix Y. Feng^2,4,6,7^, Kevan M. Shokat^1,8^

^1^Department of Cellular and Molecular Pharmacology, University of California, San Francisco, San Francisco, CA 94158, USA

^2^Helen Diller Family Comprehensive Cancer Center, University of California, San Francisco, San Francisco, CA 94158, USA

^3^Preclinical Therapeutics Core, University of California San Francisco, San Francisco, CA 94158, USA

^4^Department of Urology, University of California, San Francisco, San Francisco CA 94143, USA

^5^Department of Medicine and Division of Hematology/Oncology, University of California, San Francisco, San Francisco, CA 94158, USA

^6^Department of Radiation Oncology, University of California, San Francisco, San Francisco, CA 94143, USA

^7^Department of Medicine, University of California, San Francisco, San Francisco, CA 94143, USA

^8^Howard Hughes Medical Institute, University of California, San Francisco, San Francisco, CA 94143, USA.

**Supplementary Figures and Legends**





**Supplementary Figure S1. Androgen regulates transcription at the AR genomic locus in VCaP cells (A)** VCaP cells cultured in RPMI1640 supplemented with 5% CSS for 48h were treated for 24h with serial dilutions of DHT and immunoblotted for AR and AR-V7 protein, or **(B)** treated in three biological replicates and analyzed by RT-qPCR for AR and AR-V7 mRNA transcript levels. Data are mean ±SD. All mRNA expression levels are relative to GAPDH and normalized to the response for DHT.

**

**

**Supplementary Figure S2. (A)** VCaP cells cultured in RPMI1640 supplemented with 5% CSS for 48h, pre-treated with 10nM DHT for 24h, and then washed out and treated as indicated in three biological replicates for an additional 24h and analyzed by RT-qPCR for AR and AR-V7 mRNA transcript levels. Data are mean ±SD. P-values are relative to MDV (*** = p-value < 0.0001). All mRNA expression levels are relative to GAPDH and normalized to the response for DHT. **(B)** VCaP cells cultured in RPMI1640 supplemented with 5% CSS for 48h, pre-treated with 10nM DHT for 24h, and then treated for an additional 24h and immunoblotted for total PKC. **(C)** VCaP cells cultured in RPMI1640 supplemented with 5% CSS for 48h, and then treated with DHT or MDV in three biological replicates for 24h and analyzed in a MIB column strategy. **(D)** VCaP cells cultured in RPMI1640 supplemented with 5% CSS for 48h and then treated as indicated for 24h. Samples (N = 2 biological replicates) processed according to the Zymo-Spin ChIP Kit with LSD1 and rabbit IgG antibodies. The antibody precipitated chromatin was de-crosslinked, purified and analyzed by qRT-PCR using the primers against the regions indicated.





**Supplementary Figure S3. (A)** VCaP cells cultured in RPMI1640 supplemented with 5% CSS for 48h, pre-treated with 10nM DHT for 24h, then washed out, treated as indicated for an additional 24h and immunoblotted for AR and AR-V7. **(B)** VCaP cells cultured in RPMI1640 supplemented with 5% CSS for 48h, pre-treated with 10nM DHT for 24h, then washed out, treated as indicated for an additional 24h and immunoblotted for AR and AR-V7. **(C)** 22RV1 cells cultured in RPMI1640 supplemented with 5% CSS for 48h and then treated as indicated in three biological replicates for an additional 24h and analyzed by RT-qPCR for AR and AR-V7 mRNA transcript levels. Data are mean ±SD. P-values are relative to vehicle (*** = p-value < 0.0001). All mRNA expression levels are relative to GAPDH and normalized to the response for DHT. **(D)** 22RV1 cells cultured in RPMI1640 supplemented with 5% CSS for 48h and then treated as indicated for an additional 24h and immunoblotted for AR and AR-V7. **(E)** VCaP cells cultured in RPMI1640 supplemented with 5% CSS for 48h were treated with vehicle, 10nM DHT, 5μM MDV, 10μM Enzastaurin, or 5μM MDV + 10μM Enzastaurin in three biological replicates for 24h and then analyzed by RT-qPCR for mRNA transcript levels of DHT-AR activated genes. Data are mean ±SD. All mRNA expression levels are relative to GAPDH and normalized to the response for DMSO.





**Supplementary Figure S4. (A)** LNCaP cells cultured in RPMI1640 supplemented with 5% CSS for 48h were treated in three biological replicates for 24h as indicated and analyzed by RT-qPCR for AR and AR-V7 mRNA transcript levels. All mRNA expression levels are relative to GAPDH and normalized to the response for vehicle. Data are mean ±SD. P-values are relative to vehicle (*** = p-value < 0.0001) **(B)** LNCaP cells cultured in RPMI1640 supplemented with 5% CSS for 48h were treated as indicated for 24h and then immunoblotted for AR and AR-V7. **(C)** LNCaP cells do not have AR-V7 protein and contain LBD mutations. LNCaP (1500 cells/well) cells were cultured in RPMI1640 supplemented with 5% CSS for 48h in 96 well plates. In the presence of 0.1nM DHT, cells were treated with a nine-point threefold dilution series of MDV (beginning at 150μM) in presence or absence of a constant concentration of Enzastaurin for three days. viability subsequently measured in a CellTiter-Glo bioluminescence assay. Data are mean ±SD (N = 3 biological replicates).





**Supplementary Figure S5. (A)** Tolerability of the MDV + Enzastaurin combination arm assessed in mice in a small study and body weight measurements were tracked. Tolerability assessed at a higher dose for Enzastaurin than that chosen for evaluation in the VCaP xenograft therapeutic study. **(B).** VCaP xenograft lysates were analyzed by SDS Page and immunoblotted for total AR, AR-V7, PKCβ1, phospho-H3T6 and Histone H3. All immunoblots were processed simultaneously and normalized to Vehicle lane 1.

**

**

**Supplementary Figure S6.** Full immunoblots.
